# Supplementary material for: Quality of Care in Public County Hospitals: A Cross-Sectional Study for Stroke, Pneumonia, and Heart Failure Care in Eastern China
Source: Int J Environ Res Public Health. 2022 Jul 27;19(15):9144. doi: 10.3390/ijerph19159144 (PMC9332810; doi:10.3390/ijerph19159144)
Supplement: Supplementary file 1 [file ijerph-19-09144-s001.zip › ijerph-1810205-supplementary.pdf]

Appendix

Table S1 The detailed ICD-10 codes for included cases

**ICD-10 Codes**	**Description**
Stroke	
I63.02	Cerebral infarction due to thrombosis of basilar artery
I63.12	Cerebral infarction due to embolism of basilar artery
I63.22	Cerebral infarction due to unspecified occlusion or stenosis of basilar arteries
I65.21	Occlusion and stenosis of right carotid artery
I65.23	Occlusion and stenosis of bilateral carotid arteries
I65.29	Occlusion and stenosis of unspecified carotid artery
I63.03	Cerebral infarction due to thrombosis of carotid artery
I63.13	Cerebral infarction due to embolism of carotid artery
I63.23	Cerebral infarction due to unspecified occlusion or stenosis of carotid arteries
I63.01	Cerebral infarction due to thrombosis of vertebral artery
I63.111	Cerebral infarction due to embolism of vertebral artery
I63.21	Cerebral infarction due to unspecified occlusion or stenosis of vertebral arteries
I63.59	Cerebral infarction due to unspecified occlusion or stenosis of other cerebral artery

I63.09	Cerebral infarction due to thrombosis of other precerebral artery
I63.19	Cerebral infarction due to embolism of other precerebral artery
I63.59	Cerebral infarction due to unspecified occlusion or stenosis of other cerebral artery
I63.00	Cerebral infarction due to thrombosis of unspecified precerebral artery
I63.10	Cerebral infarction due to embolism of unspecified precerebral artery
I63.20	Cerebral infarction due to unspecified occlusion or stenosis of unspecified precerebral arteries
I63.29	Cerebral infarction due to unspecified occlusion or stenosis of other precerebral arteries
I66.0	Occlusion and stenosis of middle cerebral artery
I66.1	Occlusion and stenosis of anterior cerebral artery
I66.3	Occlusion and stenosis of cerebellar arteries
I63.32	Cerebral infarction due to thrombosis of anterior cerebral artery
I63.33	Cerebral infarction due to thrombosis of posterior cerebral artery
I63.34	Cerebral infarction due to thrombosis of cerebellar artery
I63.39	Cerebral infarction due to thrombosis of other cerebral artery
I63.6	Cerebral infarction due to cerebral venous thrombosis, nonpyogenic
I63.4	Cerebral infarction due to embolism of unspecified cerebral artery

I63.42	Cerebral infarction due to embolism of anterior cerebral artery
I63.43	Cerebral infarction due to embolism of posterior cerebral artery
I63.44	Cerebral infarction due to embolism of cerebellar artery
I63.50	Cerebral infarction due to occlusion or stenosis of cerebral artery
I63.52	Cerebral infarction due to unspecified occlusion or stenosis of anterior cerebral artery
I63.53	Cerebral infarction due to unspecified occlusion or stenosis of posterior cerebral artery
I63.54	Cerebral infarction due to unspecified occlusion or stenosis of cerebellar artery
I63.59	Cerebral infarction due to unspecified occlusion or stenosis of other cerebral artery
I63.8	Other cerebral infarction
I63.9	Cerebral infarction, unspecified
I67.89	Other cerebrovascular disease
Pneumonia	
J13	Pneumonia due to Streptococcus pneumoniae
J18.1	Lobar pneumonia, unspecified organism
J15.0	Pneumonia due to Klebsiella pneumoniae
J15.1	Pneumonia due to Pseudomonas
J14	Pneumonia due to Hemophilus influenzae
J15.4	Pneumonia due to other streptococci

J15.3	Pneumonia due to streptococcus, group B
J15.20	Pneumonia due to staphylococcus, unspecified
J15.211	Pneumonia due to Methicillin susceptible Staphylococcus aureus
J15.212	Pneumonia due to Methicillin resistant Staphylococcus aureus
J15.29	Pneumonia due to other staphylococcus
J15.5	Pneumonia due to Escherichia coli
J15.6	Pneumonia due to other aerobic Gram- negative bacteria
A48.1	Legionnaires' disease
J15.8	Pneumonia due to other specified bacteria
J15.9	Unspecified bacterial pneumonia
J15.7	Pneumonia due to Mycoplasma pneumoniae
J16.0	Chlamydial pneumonia
J16.8	Pneumonia due to other specified infectious organisms
J18.0	Bronchopneumonia, unspecified organism
J18.8	Other pneumonia, unspecified organism
J18.9	Pneumonia, unspecified organism
Heart failure	
I11.0	Hypertensive heart disease with heart failure
I50.9	Heart failure, unspecified
I13.0	Hypertensive heart and chronic kidney disease with heart failure and stage 1 through stage 4 chronic kidney disease, or unspecified chronic kidney disease

N03.9	Chronic nephritic syndrome with unspecified morphologic changes
I13.2	Hypertensive heart and chronic kidney disease with heart failure and with stage 5 chronic kidney disease, or end stage renal disease
I50.20	Unspecified systolic (congestive) heart failure
I50.21	Acute systolic (congestive) heart failure
I50.22	Chronic systolic (congestive) heart failure
I50.23	Acute on chronic systolic (congestive) heart failure
I50.30	Unspecified diastolic (congestive) heart failure
I50.31	Acute diastolic (congestive) heart failure
I50.32	Chronic diastolic (congestive) heart failure
I50.33	Acute on chronic diastolic (congestive) heart failure
I50.40	Unspecified combined systolic (congestive) and diastolic (congestive) heart failure
I50.41	Acute combined systolic (congestive) and diastolic (congestive) heart failure
I50.42	Chronic combined systolic (congestive) and diastolic (congestive) heart failure
I50.43	Acute on chronic combined systolic (congestive) and diastolic (congestive) heart failure
I50.1	Left ventricular failure
